# Small Cell Carcinoma of the Gallbladder

**DOI:** 10.7759/cureus.38444

**Published:** 2023-05-02

**Authors:** Snehasis Das, Sagar Prakash, Oseen Shaikh, Abhinaya Reddy, Uday Kumbhar

**Affiliations:** 1 Surgery, Jawaharlal Institute of Postgraduate Medical Education and Research, Puducherry, IND

**Keywords:** chromogranin, synaptophysin, gallbladder carcinoma, neuroendocrine tumor, small cell carcinoma

## Abstract

Primary neuroendocrine carcinoma of the gallbladder (GB) is a rare, highly dismal lethal disease with a fatal prognosis. A 45-year-old female presented with right upper abdomen pain and multiple vomiting episodes. Imaging studies showed diffuse thickening of the wall of the GB with locoregional invasion into the nearby structures with extensive abdominal lymph node metastasis and arteriovenous encasements. Ultrasound-guided fine-needle aspiration was done, which was diagnostic of small cell carcinoma of the GB. The patient was planned for palliative chemotherapy. A small cell variant of neuroendocrine carcinoma of the GB is a rare entity with a moribund lethality associated with it. Patients are diagnosed in advanced stages with not many treatment modalities to offer. Usually, patients are treated with palliative chemotherapy.

## Introduction

The primary neuroendocrine tumor (NET) of the gallbladder (GB) is a rare type of gastrointestinal neuroendocrine tumor [1]. These are high-grade tumors, with 75% being poorly differentiated, with a fatal prognosis. Such high-grade neuroendocrine tumors are called neuroendocrine carcinoma, which includes small and large cell carcinoma. Patients usually present right upper abdominal pain with extensive infiltration into the adjoining hepatobiliary structures, especially the liver and the GB fossa, and spread to abdominal lymph nodes. Imaging studies usually direct toward the diagnosis. The only definitive diagnosis is by histopathology or cytological examination of the tissue. The immunohistochemical examination is beneficial in diagnosing neuroendocrine tumors and helps to grade them [2]. Limited treatment options are available for NET of the GB as most of the NET are locally advanced or metastatic by the time they are diagnosed. Surgical treatment is rarely helpful as they have extensive infiltration of vital structures. Chemotherapy is the usual treatment modality for small cell variants of neuroendocrine carcinoma. We present a 45-year-old female diagnosed with metastatic neuroendocrine carcinoma of the GB, managed with palliative chemotherapy.

## Case presentation

A 45-year-old female presented with complaints of upper abdominal pain, vomiting, and fever for one week. The abdomen pain was the colicky type, mild to moderate in severity. The patient complained of repeated episodes of vomiting, around three to four times per day, mainly containing food particles. The patient also gave a history of fever for two weeks, on and off, low grade, relieved with medications. There was no history of jaundice, loss of weight, or appetite. On examination, vitals were stable. Abdominal examination revealed mild tenderness in the right hypochondrium. A vague mass was palpable in the right hypochondrium, with 3 cm hepatomegaly below the subcostal margin. Examination of other systems was within normal limits.

Blood investigations showed hemoglobin of 8.3 g/dl (13-17 g/dl) and a normal peripheral cell count. The liver function test and renal function test were within normal limits. Total bilirubin was 1.07 mg/dl (0.8-1 mg/dl), and direct bilirubin was 0.52 mg/dl (0.03-0.18 mg/dl). Tumor markers like carbohydrate antigen 125 (CA 125) were 444.3 U/mL [0-35 U/ml], carcinoembryonic antigen (CEA) was 2.7 ng/mL (0-2.5 ng/mL), and carbohydrate antigen 19-9 (CA 19-9) was 64.7 U/ml (0-37 U/ml). USG of the abdomen was done, which revealed a 10.6 cm x 6.4 cm ill-defined heterogeneous hypoechoic lesion with minimal internal vascularity noted involving the wall of the fundus of GB and adjacent liver parenchyma with minimal central intrahepatic biliary radical dilatation (IHBRD). In addition, 1.5 cm calculus was seen in the GB. The common bile duct (CBD) was dilated with a caliber of 8.5 mm, and multiple hypoechoic periportal nodes with lost fatty hilum were present.

**Figure 1:**
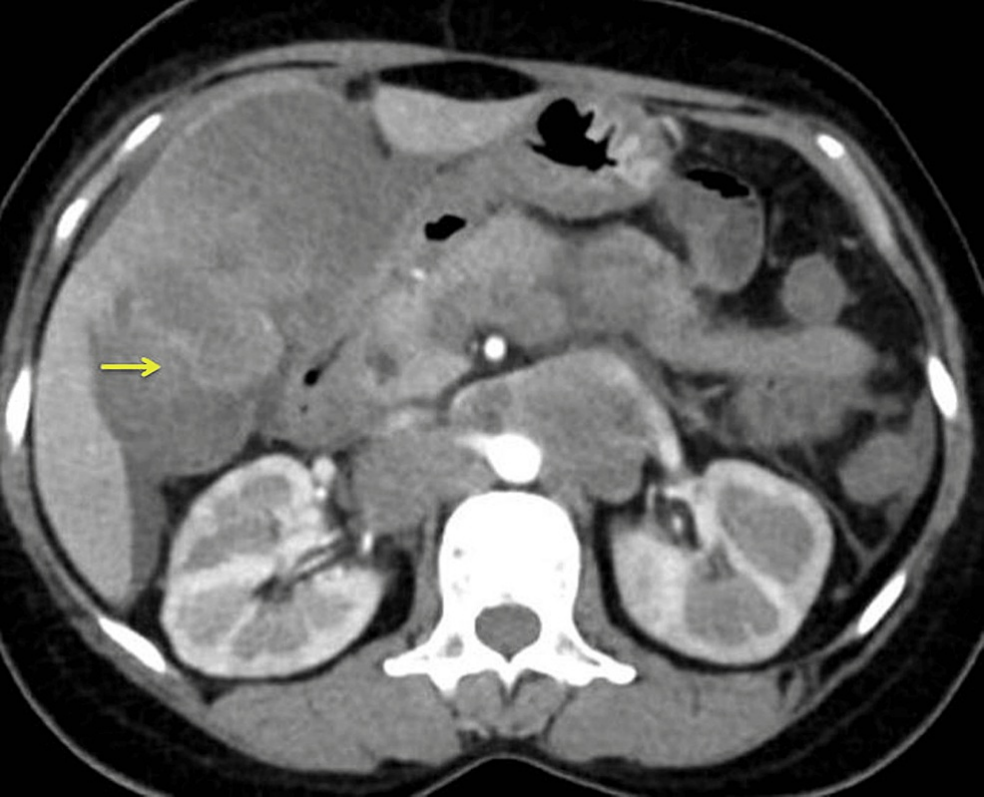
CT of the abdomen shows a large heterogeneous mass in the GB fossa (arrow)

Contrast-enhanced CT of the abdomen and thorax revealed an ill-defined heterogeneously enhancing dense, soft tissue mass arising from the body of the GB, measuring 6 x 7.1 x 6.6 cm with loss of fat planes with the stomach, duodenum, left lobe of the liver, anterior surface of right kidney, and hepatic flexure and proximal transverse colon (Figure 1).

**Figure 2:**
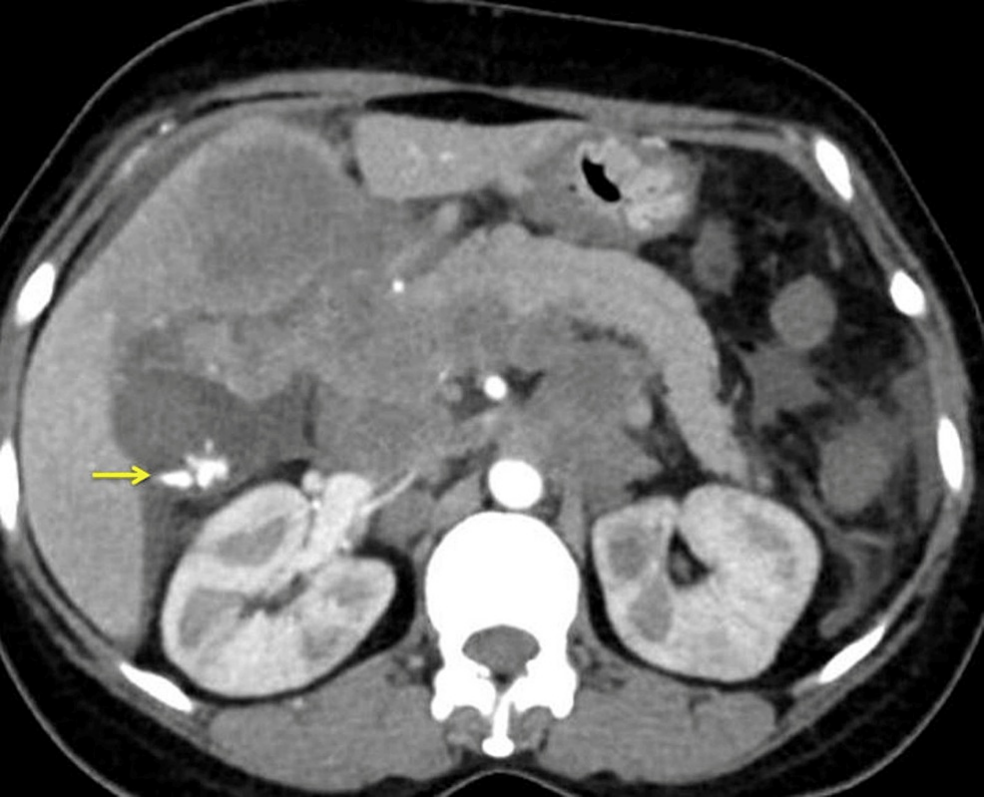
CT of the abdomen showing cholelithiasis (arrow)

The proximal CBD encasement was seen with proximal CBD measuring 6 mm with minimal central IHBRD. There was also evidence of cholelithiasis (Figure 2).

**Figure 3:**
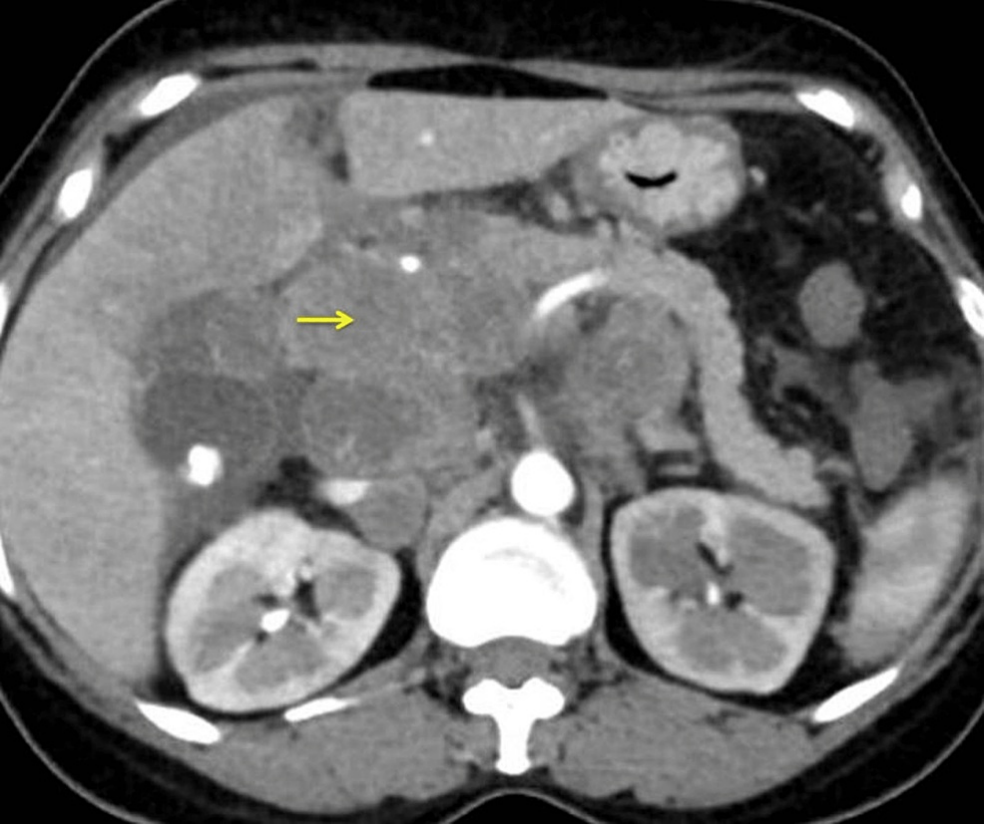
CT of the abdomen showing conglomerated lymph nodal mass near the celiac axis (arrow)

Multiple enlarged heterogeneously enhancing nodes with non-enhancing areas (necrosis) within are seen in the peri-portal, para-aortic, para-caval, and aortocaval regions and along the branches of celiac and superior mesenteric trunks. The nodes in the retroperitoneum encase bilateral renal arteries with no narrowing. The nodes along the celiac axis are confluent and encase the distal celiac artery and the origins of the left gastric, splenic, and common hepatic arteries (Figure 3).

**Figure 4:**
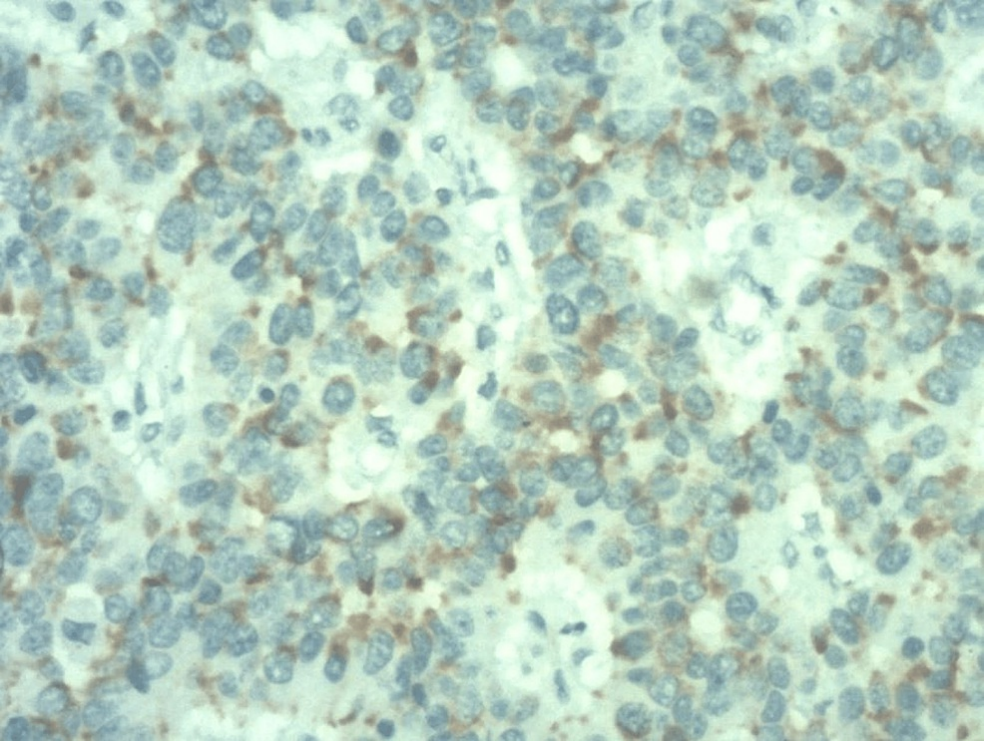
Immunohistochemistry image showing diffuse synaptophysin-positive cells (synaptophysin staining, 20X resolution)

USG-guided fine needle aspiration (FNA) from the lesion in the GB fossa was highly cellular. It showed predominantly scattered atypical cells displaying moderately pleomorphic nuclei with irregular nuclear membrane, finely dispersed chromatin, few cells showing prominent nucleoli, and moderate cytoplasm. Few abnormal cells and multinucleated cells were also seen. There was an increase in mitotic activity. There was a prominent perivascular arrangement of tumor cells. Foci of necrosis were seen. The tumor cells show diffuse positivity for synaptophysin (Figure 4).

Few cells were also positive for cluster of differentiation (CD) 56 and focal positivity for chromogranin A and Pan-cytokeratin on immunohistochemistry. The tumor cells were negative for leukocyte common antigen (LCA), cytokeratin 7 (CK7), CD30, CD3, and CD79a. The proliferation index (Ki-67) was 90%.

The patient was diagnosed to have locally advanced neuroendocrine carcinoma of the GB. The patient was discussed in a multidisciplinary tumor board and was planned palliative chemotherapy (oxaliplatin and etoposide) and the best supportive care. The patient was also registered in the palliative pain clinic for pain management.

## Discussion

The primary NET of the gastrointestinal tract is uncommon. The most common sites are usually the appendix, jejunum, and rectum. The other sites include the duodenum, colon, and stomach. Regarding metastatic sites, lymph nodes are the most common, followed by the liver, lungs, and peritoneum. Exceptionally, the GB was postulated to be the rare site for a NET in the gastrointestinal tract [2]. The NET of the GB corresponds to about 2.1% of all GB tumors [3].

The origin of a GB neuroendocrine neoplasm is still controversial, with no solid evidence in the literature. Most scientists have postulated epithelial metaplasia with concurrent inflammation as the major driving factor due to the association of most NET with cholelithiasis. In general, there is not a single neuroendocrine cell in the GB. However, it has been documented in the resected GB of cholelithiasis patients that the mucosa of the GB may have intestinal or gastric metaplasia [4,5].

Another school of thought coexists, theorizing the origin of GB NET from adenocarcinoma cells in direct transition. This theory is intriguingly possible with similar coexisting hybrid tumors in literature [6]. In addition, a series of less than 50 reported cases have postulated heterotopic pancreatic tissue in the GB as a potential carcinogen for the organ [7]. From a genetic angle, no association with other traits has been established except for a single patient with a clear cell GB NET with Von Hippel-Lindau disease [7].

Most of the patients present with a vague right upper pain abdomen with distension which might mimic stone disease. No specific signs exist. Functionally, GB neuroendocrine carcinoma can be divided into secretory and non-secretory types based on the production of peptides by the tumor. The non-secretory one usually presents with signs of malignancy. At the same time, the functional tumors might show a definite syndromic presentation mimicking carcinoid syndrome, leading to diarrhea, flushing, edema, and wheezing [8].

Patients undergo radiological studies initially in the form of a CT abdomen and pelvis with baseline ultrasonography of the abdomen to stage the disease and assess possible surgical resectability [9]. In addition, patients undergo positron emission tomography CT to detect any possible distant metastasis. MRI would be the modality of choice, and, in combination with a magnetic resonance cholangiopancreatography, would report sensitivities of nearly 100% for staging and invasion assessment [10]. Nonetheless, imaging modalities cannot differentiate a GB adenocarcinoma from a NET. Hence, histological, cytological, and immunohistochemical analyses help to arrive at a definitive diagnosis. A guided biopsy or FNA is usually done to conclude the diagnosis. Immunohistochemical staining is done for confirmation, as a histopathological examination is the only definitive way to ascertain the diagnosis [3]. Our patient's growth had infiltrated the liver with para-aortic lymphadenopathy encasing all major intra-abdominal arteries.

NET has been classified by the WHO based on several parameters. It comprised grading from G1-G3 and differentiation sequence based on mitotic figures and Ki-67 staining positivity. These include includes well-differentiated and poorly differentiated tumors. The well-differentiated tumors were divided into G1 (low grade) and G2 (intermediate grade). The poorly differentiated NET (mitotic figures > 20/10 high power field and Ki-67 staining > 20% positive) were graded as G3. Poorly differentiated variety is again classified as large cell carcinoma, small cell carcinoma, and mixed adenocarcinoma neuroendocrine carcinoma [11]. Nearly 75% of the NET will stain positive for synaptophysin, followed by around 20% for chromogranin A [12]. The tumor was positive for synaptophysin and chromogranin A, with a histology diagnostic of a small cell variant of neuroendocrine carcinoma.

Gold-standard treatment for a GB NET involves en bloc surgical resection. A simple cholecystectomy would suffice for early-stage carcinomas (Tis and T1). An advanced disease would warrant an aggressive radical cholecystectomy with segmental hepatic resection and regional lymphadenectomy with clear tumor-free margins [13,14]. The median survival period has been documented to be less than a year [8]. The use of chemotherapy as an adjunct to surgery is still doubtful in its usefulness, as there was no evident increase in the median survival periods after adjuvant chemotherapy [14]. However, it has been deemed the best form of palliation. The effectiveness of adjuvant radiotherapy is unclear as, traditionally, the NET is insensitive to radiotherapy. Despite a morbid aggressive surgical approach, most candidates for supposed curative resections also develop recurrent metastatic disease. Overall, the prognosis remains fatal as the disease moves silently. By the time it is diagnosed, it is usually an advanced disease with no significant response to any form of treatment [14]. Our patient was also deemed metastatic and offered palliative chemotherapy with pain management.

## Conclusions

Small cell variant of neuroendocrine carcinoma of the GB is the rarest type of tumor. It has a poorer prognosis as compared to any other gastrointestinal neuroendocrine carcinoma. Extensive hepatobiliary structure infiltration and nodal dissemination in the initial scans should raise suspicion of neuroendocrine carcinoma. A multimodal salvage surgery followed by a chemo-radiotherapy approach is seen to give the best response. For patients with limited disease, surgical treatment may be helpful. However, the chances of recurrence following surgical excision are still high. With all treatment options available, the diagnosis of neuroendocrine carcinoma of GB carries a poor prognosis, and survival among these patients is usually six months to one year.

## References

[1] Ahmed M (2020). Gastrointestinal neuroendocrine tumors in 2020. World J Gastrointest Oncol.

[2] Zou YP, Li WM, Liu HR, Li N (2010). Primary carcinoid tumor of the gallbladder: a case report and brief review of the literature. World J Surg Oncol.

[3] Niu C, Wang S, Guan Q, Ren X, Ji B, Liu Y (2020). Neuroendocrine tumors of the gallbladder. Oncol Lett.

[4] Yadav R, Jain D, Mathur SR, Iyer VK (2016). Cytomorphology of neuroendocrine tumours of the gallbladder. Cytopathology.

[5] Singh R, Balasubramanian I, Zhang L, Gao N (2020). Metaplastic Paneth cells in extra-intestinal mucosal niche indicate a link to microbiome and inflammation. Front Physiol.

[6] Frizziero M, Chakrabarty B, Nagy B, Lamarca A, Hubner RA, Valle JW, McNamara MG (2020). Mixed neuroendocrine non-neuroendocrine neoplasms: a systematic review of a controversial and underestimated diagnosis. J Clin Med.

[7] Lee SW, Yun SP, Seo HI (2013). Heterotopic pancreas of the gallbladder associated with segmental adenomyomatosis of the gallbladder. J Korean Surg Soc.

[8] Chen C, Wang L, Liu X, Zhang G, Zhao Y, Geng Z (2015). Gallbladder neuroendocrine carcinoma: report of 10 cases and comparision of clinicopathologic features with gallbladder adenocarcinoma. Int J Clin Exp Pathol.

[9] Lee SE, Kim KS, Kim WB (2014). Practical guidelines for the surgical treatment of gallbladder cancer. J Korean Med Sci.

[10] Edge SB, Compton CC (2010). The American Joint Committee on Cancer: the 7th edition of the AJCC cancer staging manual and the future of TNM. Ann Surg Oncol.

[11] La Rosa S, Marando A, Sessa F, Capella C (2012). Mixed adenoneuroendocrine carcinomas (MANECs) of the gastrointestinal tract: an update. Cancers (Basel).

[12] Perren A, Couvelard A, Scoazec JY (2017). ENETS consensus guidelines for the standards of care in neuroendocrine tumors: pathology: diagnosis and prognostic stratification. Neuroendocrinology.

[13] Kim J, Lee WJ, Lee SH (2011). Clinical features of 20 patients with curatively resected biliary neuroendocrine tumours. Dig Liver Dis.

[14] Eltawil KM, Gustafsson BI, Kidd M, Modlin IM (2010). Neuroendocrine tumors of the gallbladder: an evaluation and reassessment of management strategy. J Clin Gastroenterol.

